# Minimal Design Principles for Icosahedral Virus Capsids

**DOI:** 10.1021/acsnano.1c04952

**Published:** 2021-09-07

**Authors:** Manuel Martín-Bravo, Jose M. Gomez Llorente, Javier Hernández-Rojas, David J. Wales

**Affiliations:** † Departamento de Física and IUdEA, Universidad de La Laguna, 38205 Tenerife, Spain; ‡ Department of Chemistry, University of Cambridge, Lensfield Road, Cambridge CB2 1EW, United Kingdom

**Keywords:** virus capsids, icosahedral shells, optimal packing, cost functions, basin-hopping

## Abstract

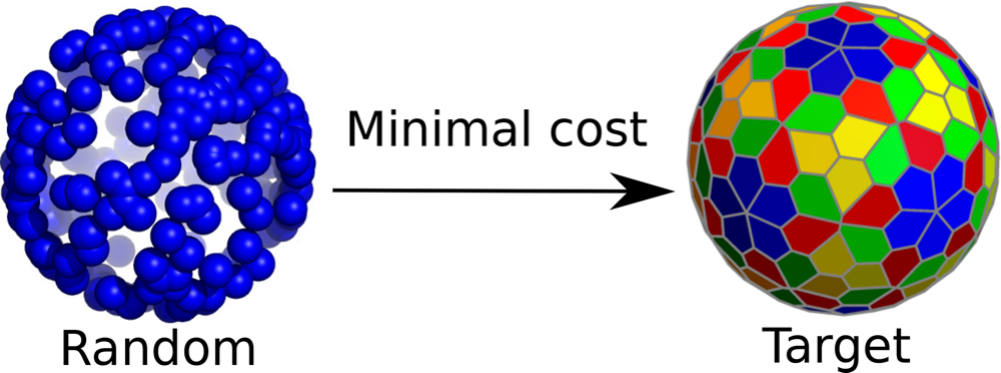

The geometrical structures of single- and multiple-shell icosahedral virus capsids are reproduced as the targets that minimize the cost corresponding to relatively simple design functions. Capsid subunits are first identified as building blocks at a given coarse-grained scale and then represented in these functions as point particles located on an appropriate number of concentric spherical surfaces. Minimal design cost is assigned to optimal spherical packings of the particles. The cost functions are inspired by the packings favored for the Thomson problem, which minimize the electrostatic potential energy between identical charged particles. In some cases, icosahedral symmetry constraints are incorporated as external fields acting on the particles. The simplest cost functions can be obtained by separating particles in disjoint nonequivalent sets with distinct interactions, or by introducing interacting holes (the absence of particles). These functions can be adapted to reproduce any capsid structure found in real viruses. Structures absent in Nature require significantly more complex designs. Measures of information content and complexity are assigned to both the cost functions and the capsid geometries. In terms of these measures, icosahedral structures and the corresponding cost functions are the simplest solutions.

Viruses are perhaps the simplest natural organisms capable of self-assembly, self-replication, and other biological functions, always within a host living cell. Their structure and behavior are encoded in the virion genetic material (DNA or RNA), which is enclosed in a protein coat known as a capsid. In most viruses, this capsid is approximately icosahedral. The corresponding point group symmetries are *I* and *I*_*h*_, the latter having an inversion center of symmetry. *I* is the highest symmetry that a system composed of a finite number of asymmetric constituent elements, such as proteins, can have, and therefore represents the approximate symmetry of icosahedral viral capsids at the atomic scale. However, when the structure is coarse-grained, both *I* and *I*_*h*_ symmetries are possible.

It is well-known that the icosahedral design has many advantages, such as high stability, maximal storage capacity, and minimal information coding requirements.^1^ This information simplicity has led to general principles to geometrically classify icosahedral capsids. Among these rules, the quasi-equivalence principle of Caspar and Klug (CK)^2^ laid the foundations for modern structural virology. These authors proposed a geometrical model of the capsid in terms of repeating subunits (capsomers, both pentamers and hexamers), by folding a 2D hexagonal lattice, where the sites are identified by a pair of non-negative integers (*h* and *k*). The resulting icosahedral shell is made of 20 equilateral triangular faces, whose area, expressed in units of the smallest icosahedron that can be built, is given by the triangulation number *T* = *h*^2^ + *k*^2^ + *kh*. Thus, the possible values for *T* are discretized, *i.e., T* = 1, 3, 4, 7, ..., and are used as a structural classification for most icosahedral viruses. Within this scheme, an icosahedral shell with triangulation number *T* comprises 12 pentamers and 10(*T* – 1) hexamers, yielding a total of 10*T* + 2 capsomers and 60*T* capsid proteins. The 12 pentamers correspond to the 12 dislocations required to form a convex icosahedral cage. These rules have been adapted to accommodate prolate nonicosahedral capsids.^3−6^

Generalizations of CK geometrical construction have recently been provided to account for other anomalous icosahedral viral structures.^7,8^ By applying the CK construction to the other three 2D Archimedean hexagonal lattices, namely trihexagonal, snub hexagonal, and rhombitrihexagonal and their duals, Twarock and Luque^8^ were able to represent anomalous capsid architectures. These structures combine major and minor coat proteins to produce totals excluded by the CK construction. Viruses in the HK97 lineage provide examples of this construction from the trihexagonal lattice. This scheme provides an explanation for alternative capsid layouts with identical stoichiometry in other viral lineages. However, examples corresponding to the regular rhombitrihexagonal and snub hexagonal lattices are currently unknown.^8^

The scheme of Rochal and co-workers^7^ uses the same CK map from the 2D hexagonal lattice onto the icosahedron and defines a corresponding spherical lattice, which is denoted as ⟨*h,k*⟩ in terms of the CK indices. The protein positions in the planar structure are chosen by analyzing compatibility with the spherical lattice positions, which may be different from those chosen in the original CK model. Specifically, the nodes in the icosahedral spherical lattice are classified according to their local symmetry as trivial (a multiple of 60), 2-fold (just 30), 5-fold (just 12), and 3-fold (just 20), respectively. Proteins, as asymmetric units, can only be assigned to trivial nodes without local symmetry. Symmetric nodes require compatible symmetric subunits made of several proteins. This model adds other capsid structures to those provided by the CK model. The approach can also be used to account for complex double-shelled capsids by introducing a notion of commensurability.^7,9−12^ The Landau theory of crystallization applied to spherical icosahedral lattices in a previous publication by Lorman and Rochal^9^ was also considered for the anomalous structures. This scheme uses an expansion of the critical density deviation in a reduced basis set of spherical harmonics adapted to the icosahedral symmetry (icosahedral harmonics). Further details of all these geometrical construction schemes are summarized and analyzed in recent reviews by Zandi *et al.*(13) and Šiber.^14^

The 2D hexagonal lattice is ubiquitous in Nature, exhibiting maximal symmetry, optimal packing, and optimal covering. A functional of any of these properties may be designed so that, when minimized, it will provide as its target the hexagonal packing. In this work, we will refer to such functionals as design or cost functions. Hence, cost functions whose targets are the 2D hexagonal lattice are the simplest ones to define for periodic structures in two dimensions. On the other hand, 2D packings, such as square close-packing, will require more complex designs. This situation highlights a direct relationship between the complexity of the cost function and the relative abundance of their targets in nature. The present contribution extends this idea to icosahedral viral capsids. We will design cost functions whose targets are the structures observed in known viral capsids. Each target structure will be reproduced as the global minimum of the corresponding cost function. These functions must satisfy the principle of minimal complexity, *i.e.*, minimal information requirements in the mathematical formulation. The diversity of icosahedral capsids then leads to a variety of minimal cost functions. The relative complexity of these functions contains relevant physical information about the corresponding capsid architectures.

The long-range order that underpins the designs must be encoded in the relatively (at the virion scale) short-range molecular interactions that drive the self-assembly of such ordered structures. There are many examples in theoretical virology that demonstrate a direct connection between order in the target structures and the minimal information required to design short-range models for the interaction between coarse-grained capsid subunits, which reproduce the target geometries.^4,6,15−50^ Hence, an important connection can be made between the complexity of the minimal cost functions and the essential components of the interaction potential required to support these target structures, preferably as global minima on the energy landscape. Some examples that illustrate this connection will be provided in this work.

Another relevant connection may be made between the complexity of cost functions and the relative abundance of the corresponding target structures in Nature: an increase in complexity corresponds to a lower probability of self-assembly for the target structure. The relative abundance of *T* = 3 and *T* = 4 structures and other observations will illustrate this connection.

The targets of our cost functions will correspond to particular distributions of given numbers of building blocks on a spherical surface. The building blocks can be structured or point particles. The CK construction scheme and the generalizations discussed above fold the 2D hexagonal lattice onto the icosahedral shells, providing optimal covering and packing. Optimal packings, coverings, and maximal convex-hull volumes on the sphere correspond to well-defined classes of optimization problem.^51^ Another way to distribute a fixed number of point particles *N* on a sphere can be derived from the well-known Thomson problem.^52,53^ In this case, the cost function corresponds to the electrostatic potential energy when a unit charge is associated with each particle. After scaling, this cost function has no adjustable parameters and, therefore, represents a scheme with minimal information requirements and thus minimal complexity. When compared with the other three optimization problems on the sphere, the Thomson problem is the one that provides the most icosahedral packings. This situation occurs for *N* = 12, 32, 72, 122, 132, 192, 212, 272, ...,^54−56^ and the minimal cost structures, when these numbers refer to capsomers, correspond to icosahedral viral capsids with *T* = 1, 3, 7, 12, 13, 19, 21, 27, ..., respectively. Therefore, these capsids can be classified straightforwardly at the capsomer coarse-graining scale. There are missing *T* values, namely *T* = 4, 9, 16, 25, 28, .... Furthermore, if particles are meant to represent single proteins, the Thomson problem fails to provide the right structures.

Our design generalizes the Thomson form to increase its predictive power and to account for more complex icosahedral structures. The symmetry constraint is incorporated through interaction of the particles with an external field, which is expanded in the symmetry adapted basis set of icosahedral harmonics (IH).^57^ The smaller the number of basis functions required to produce a given structure, the lower is its information content and complexity. The general form can also account for the structure of double-shell capsids and capsids with protruding decorations. Interestingly, complexity can be reduced by a suitable choice for the building blocks distributed on the sphere and thus depends on the coarse-grained scale applied, with monomers, dimers, trimers, pentamers, or hexamers as possible choices. Moreover, these building blocks can be holes instead of particles. Once the required terms and particle types are included in the cost function and its parameters fixed, the positions are optimized to find the global minimum.

Cost functions can be designed to reproduce practically any capsid structure, including those supported by the models of Caspar and Klug,^2^ Twarock and Luque,^8^ and Rochal and co-workers,^7,9−12^ along with many other anomalous structures. Moreover, by increasing its complexity, the model allows finer control over the positions of the particles in the final structure. Quantitative measures of information content and complexity are assigned to both the cost functions and the capsid geometries in the Conclusions section.

## The Design Function

To provide the most general form of the design function we consider a virion capsid composed of *M* spherical shells. Each shell, labeled *s*, has radius *R*_*s*_ and contains *N*_*s*_ identical particles at positions **r**_*i*_, given in terms of their spherical coordinates (*R*_*s*_, θ_*i*_, ϕ_*i*_). Orientational degrees of freedom could also be assigned to these units, but we will not consider this extension here because it increases complexity and is not required for our purposes. Different shells may share the same radius, particle number, or both. When there is more than one shell with a common radius, these should be understood as corresponding to just one actual virion shell, with separate sets of particles. The total number of particles is therefore *N* = ∑_*s*=1_^*M*^*N*_*s*_. We use *r*_*ij*_ = |**r**_*j*_ – **r**_*i*_| to denote the distance between two particles in the same or different shells. The cost function  is then
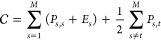
1where *P*_*s*,*s*_ and *P*_*s*,*t*_ are the contributions associated with the absolute arrangement of the units in a given shell, and between shells, respectively, and *E*_*s*_ is determined by external constraints.

*P*_*s*,*s*_ and *P*_*s*,*t*_ will be chosen to favor optimal packing and/or covering structures. There is no unique choice for these functions. For instance, Conway and Sloan^51^ describe the packing problem (maximize the minimal distance between particles), the covering problem (minimize the maximal neighbor distance), and the maximal volume problem (maximize the volume of the convex hull). These three problems lead to quite uniform distributions of points on the sphere. The Thomson problem with generalized electrostatic interactions 1/*r*_*ij*_^α^ is also an option in this field of mathematical optimization.^53^ Among its attractive properties are analyticity, easy generalization, and straightforward computational implementation. An additional property is decisive for our purposes: the Thomson problem provides the largest number of icosahedral structures compared to the other three optimization problems on the sphere.^51^ Since we aim at finding minimal designs for icosahedral capsids, the Thomson form is our choice for *P*_*s*,*t*_. Hence, we have
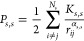
2
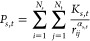
3with parameters *K*_*s*,*s*_, *K*_*s*,*t*_ = *K*_*t*,*s*_, α_*s*,*s*_, α_*s*,*t*_ = α_*t*,*s*_. *K*_*s*,*t*_ and α_*s*,*t*_ represent the strength and the range of the interaction between a particle in shell *s* and another particle in shell *t*, respectively.

The functions *E*_*s*_ are included to favor icosahedral symmetry in the capsid design. *E*_*s*_ is expressed as the interaction of the particles in the given shell with an external field of icosahedral symmetry, which is expanded in terms of the icosahedral harmonics (IH). The explicit form is
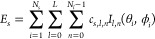
4where *c*_*s*,*l*,*n*_ are additional parameters and *I*_*l*,*n*_(θ_*i*_,ϕ_*i*_) represents the icosahedral harmonic with index *l* coming from the corresponding spherical harmonic, and index *n* running over the *n*_*l*_ linearly independent IH that can be constructed from the 2*l*+1 spherical harmonics for each *l*. These IH, which transform as the totally symmetric irreducible representation in *I*, exist for a reduced, although infinite, subset of *l* values (*e.g.*, *l* = 0, 6, 10, 12, 15, 16, 18, ...) with *n*_*l*_ = 1 for all IH up to *l* = 30. Further details are given in the Methods.

The cost function is fully defined when all its parameters are fixed, including the number of shells, the number of particles (or holes) in each shell, the parameters of the Thomson interactions, the number of IHs, and their corresponding coefficients. These values must be chosen to produce the target structure as the global minimum of the cost function. Parameters with integer values, such as the number of shells and particles, must be inferred from the target structure. Parameters with continuous values, such as Thomson interactions, can usually be varied over a wide range without significant effects on the target structure. Finally, the values of the *c*_*s*,*l*,*n*_ coefficients in the external field admit changes of around 10%, but at the same time these parameters permit a finer adjustment of the particle positions. The choice of all these parameters is determined empirically, guided by the geometry of the target structure. Once they are fixed, we find the particle coordinates (configuration) corresponding to the lowest minimum of  using basin-hopping global optimization^58,59^ to check whether that configuration indeed corresponds with the target structure. Basin-hopping steps correspond to random perturbations followed by local minimization, and an acceptance/rejection criterion of the new minimum is based on a Metropolis condition with a fixed effective temperature parameter (see the Methods).

One is free to apply the design function to actual particles (proteins or capsomers) or missing entries in the structure. Whether to use particles or holes may depend upon different factors. For instance, if the building blocks are asymmetric they cannot occupy symmetric locations (2-fold, 3-fold, and 5-fold axes). Our scheme may then require holes for that purpose. The approach taken by Rochal and co-workers^7^ explicitly excludes those symmetric locations for the asymmetric units. In other cases the holes are present in the capsid structure and may be associated with a particular biological function. The nature of such functions is outside the scope of the present work, but the observed holes are required to provide minimal cost functions.

## Results and Discussion

Whenever possible, we will use the notation of Rochal *et al.*,^7^ ⟨*h,k*⟩, to label the capsid structures, along with the *T* triangulation numbers.

Given the periodicity properties of the IH,^57^ the expansion of the real general mass density in a given icosahedral capsid may require a large number of terms.^9,10^ However, the smoothest density compatible with a given ⟨*h,k*⟩ spherical lattice can be accomplished with a minimal set of icosahedral harmonics having *l* ≤ *l*_c_, with *l*_c_(*h*,*k*) being a cutoff value that increases with both *h* and *k*. An explicit and rigorous form for *l*_c_(*h*,*k*) is not known, but values for particular lattices can be estimated. For instance for the ⟨3,0⟩ lattice we find *l*_c_(3,0) = 18. However, when we turn our attention to the IH expansion of the external field used in the cost functions we find that only a reduced number of terms (those represented in Figure 8) are required to produce the target distribution of capsid building blocks. This result is due to the dominant role of the electrostatic-like contributions in the cost function, which favors a uniform distribution, and prevents clumping of particles at the minima of the external potential.

We are not free to place a completely arbitrary number of units (or holes) on the shell; the required icosahedral symmetry of the capsid and the internal symmetry of the building blocks constrain the options (the symmetry of a hole is determined by the local symmetry of its site location). If these building blocks have no internal symmetry elements, as for proteins, they cannot lie on rotation axes. In this case, one building block implies that 59 additional symmetry equivalent copies are required; thus, only multiples of 60 are allowed. Symmetric units allowed in the icosahedral symmetry point group *I* must have at least a rotation symmetry axis with one of three possible orders: 2-fold, 3-fold, and 5-fold. The icosahedron has just 30 positions compatible with 2-fold units, 20 positions for 3-fold units, and 12 for 5-fold units. If one of these locations is occupied the complete set must be present. Therefore, we can have steps in the allowed number of units of 12, 20, and 30, corresponding to the occupation of the 5-fold, 3-fold, and 2-fold axes, respectively. The allowed values for the number of units in a shell are therefore
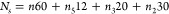
5with *n* a positive integer (or zero), with *n*_5_, *n*_3_, and *n*_2_ being either 0 or 1.

We scale the cost function by fixing the radius of the innermost shell to *R*_1_ = 1 and its strength parameter to *K*_1,1_ = 1. We will also choose the same value for all of the range parameters, so that α_*s*,*t*_ = 1.

### Distribution of Capsomers in a Single Shell

The target structures in this subsection are single-shell capsids, for which the CK rules hold, and the building blocks are initially equivalent capsomers. For these single shells, the simplest cost function corresponds to the Thomson problem with no free parameters. The icosahedral capsids with relatively high abundance in Nature that can be targeted with the simplest cost functions have triangulation numbers *T* = 1, 3, and 7. We hypothesize a connection between the abundance and the simplicity of the cost functions in these cases. Further evidence for this connection comes from comparing these abundances to those of capsids that require more complex cost functions, such as those with triangulation numbers *T* = 4 and 9 (these are the smallest capsids of the ⟨*h*,0⟩ class, which cannot be reproduced with the Thomson cost function).^55,56^ Taking into consideration the differences in size, *T* = 3 and *T* = 7 capsids still appear to be significantly more abundant that their neighbors with *T* = 4 and *T* = 9. The smallest *T* = 1 capsids are by far the most abundant among the icosahedral examples. Quantitative measures of complexity that include size effects will be discussed in the conclusions.

In the introduction, we also discussed a possible connection between complexity of the cost function and the essential components of the short-range interparticle potential required to support these target structures as ground states. Our hypothesis in this case is that interaction potential models at the capsomer coarse-grained scale will be more difficult to construct for *T* = 4 and *T* = 9 structures than for those with *T* = 3 and *T* = 7, respectively. This situation is confirmed by the calculations performed with previous coarse-grained interaction models. Some of them^16,31,47^ are unable to produce *T* = 4 (and *T* = 9) structures at all^16,31^ or require two types of subunits and additional terms in the interaction.^47^ Furthermore, all particles in the simple Thomson cost function for capsids with *T* = 1, 3, and 7 are identical, which suggests that coarse-grained short-range interaction models based on only one type of building block might be able to support these structures as global minima. This situation obviously arises for *T* = 1 capsids, which can be represented in terms of identical pentamers. Capsids with *T* = 3 and 7 require pentamers and hexamers. However, calculations based on a minimalist model and a single type of building block^47,49^ also support our previous conjecture for these capsids. There is even a natural realization of a *T* = 7 capsid with a single type of subunit, *i.e.* the papillomavirus capsid, which is composed of pentamers.^60^ Notice that 5-fold is the minimal required symmetry for identical subunits that, in a *T* = 7 capsid, must be located at 5-fold sites (12 of them) or at asymmetric sites (the other 60). If the same argument is applied to a *T* = 3 capsid built from the same capsomer type, the building block would require both 5-fold and 3-fold symmetries, which are the local symmetries of the capsomer sites in this structure. Thus, the required subunit would be at least a 15-component capsomer, which has not been observed in *T* = 3 or any other capsids.

We now focus on the cost functions required to target the ⟨*h*,0⟩ capsids not supported by the Thomson problem. Here, we introduce an external field to favor the icosahedral geometries, which makes the cost function versatile enough to support the missing structures. We have checked these results for *T* up to *T* = 28.

**Figure 1:**
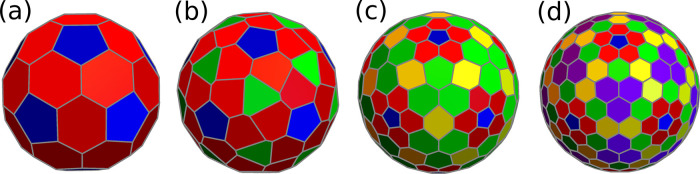
Voronoi representations of the structures obtained with the cost-function parameters given in Table 1. These structures correspond to capsids of Human Hepatitis B Virus (a, *T* = 4, ⟨2,0⟩), Enterobacteria Phage N4 (b, *T* = 9, ⟨3,0⟩), Varicella-zoster Virus (c, *T* = 16, ⟨4,0⟩) and Human Adenovirus (d, *T* = 25, ⟨4,0⟩). For each structure, elements in the same orbit are colored identically. For example, in (b) we have 12 pentagons with site symmetry *C*_5_, 20 green hexagons with site symmetry *C*_3_, and 60 red asymmetric hexagons.

In Table 1 we present example parameter values used in  for some of these shells, where we have dropped the *s* subscript in the coefficients *c*_*s,l,n*_ since we are dealing with a single shell. The structures are presented in Figure 1, using Voronoi tessellation diagrams. Examples are known for Human Hepatitis B Virus (*T* = 4),^61^ Enterobacteria Phage N4 (*T* = 9),^62^ Varicella-zoster Virus (*T* = 16),^63^ and Human Adenovirus (*T* = 25).^64^ We see from this figure that the identical units of the model separate in different symmetry equivalent classes (orbits of the point group) that are determined by their position on the spherical lattice. These classes are shown with different colors in each of the shells illustrated in Figure 1.

**Table 1:** Example Values of the Coefficients *c*_*l*,*n*_ in the Expansion of the External Field in IH^a^

*T*	*N*	*c*_6,0_	*c*_10,0_	*c*_12,0_	*c*_15,0_
4	42	0.3	0.1	–1.5	0
9	92	1.3	–0.8	–1	0.5
16	162	–10	0	0	0
25	252	–10	0	0	0

a *N* gives the number of capsomers on the shell.

The set of elements of a group  that leave any point, *s*, unchanged define a subgroup , also known as the stabilizer, or site symmetry, of the point. The other members of the orbit are defined by the  cosets of , and Lagrange’s theorem tells us that  is an integer divisor of . The possible orbit sizes are therefore , where  is any subgroup of . For orbits consisting of point particles on a sphere of finite radius only the subgroups corresponding to stabilizer groups for a single vertex are possible, *i.e.*, *C*_5_, *C*_3_, *C*_2_, and *C*_1_ in *I*, giving orbit sizes 12, 20, 30, and, finally, 60 for a particle that does not lie on any symmetry elements. Of course, these values are consistent with eq 5. In *I*_*h*_, the relevant subgroups are *C*_5*v*_, *C*_3*v*_, *C*_2*v*_, *C*_*s*_, and *C*_1_, so the only additional orbit size is 120.

The components of repeating units in the Caspar−Klug construction are inequivalent if they are not related by symmetry, but the repeating asymmetric units themselves form an orbit of the icosahedral point group.

**Figure 2:**
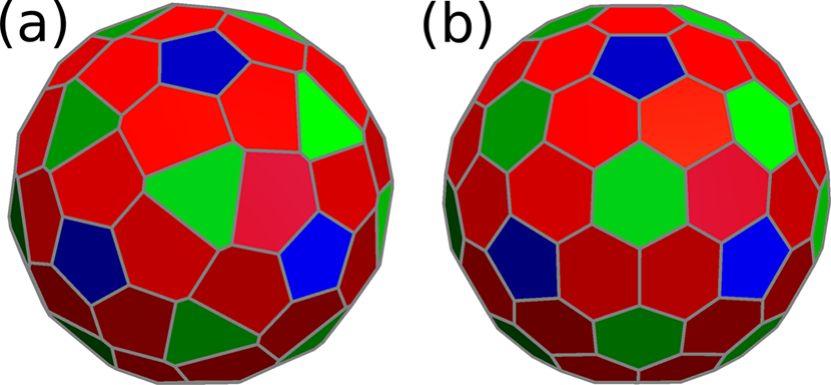
Voronoi representations of a *T* = 9 capsid obtained with the cost-function parameters given in Table 2. Case (a) is the anomalous prediction and case (b) is the CK structure with spherical lattice indices ⟨3,0⟩.

The shell (b) with triangulation number *T* = 9 is anomalous as far as the CK rules are concerned. Unlike the other shells in Figure 1, it does not have an inversion center and does not seem to match the structure of the Enterobacteria Phage N4 virus capsid, which exhibits a center of inversion at the capsomer coarse-graining scale and, thus, follows the CK scheme. Within our model, it is still possible to achieve this missing *I*_*h*_ symmetry by increasing the complexity of the cost function to include a higher *l* IH, namely *l* = 18, which coincides with the cutoff value *l*_c_(3,0) = 18 estimated for this spherical lattice (⟨3,0⟩) at the beginning of this section. Another possibility is the separation of the 92 capsomers into two inequivalent sets (two different shells with the same radius) and introduction of two different strength parameters *K*_1,2_ = *K*_2,1_ and *K*_2,2_. The two sets of parameter values given in Table 2 produce the *I* (a) and *I*_*h*_ (b) structures, respectively, which are shown in Figure 2. However, while the two-shell alternative for the *I* structure supports the laevo and dextro isomers as degenerate minima of the cost function, the single-shell cost function without the *l* = 18 IH component can select one or the other by changing the sign of the *c*_15,0_ coefficient corresponding to the odd IH, which breaks the inversion symmetry.

**Table 2:** Example Parameter Values for the Two-Shell Cost Functions with Anomalous (a) and Normal (b) *T* = 9 Structures as Target Structures

case	*N*_*s*_	*R*_*s*_	*K*_*s*,*t*_
(a)			
(b)			

### Distribution of Proteins in a Single Shell

The target structures in this subsection are those of single-shell capsids with single proteins as building blocks. Specifically, we will consider the capsids of Satellite Tobacco Mosaic Virus (STMV),^65^ L-A Virus,^66^ Dengue Virus,^67^ Cowpea Chlorotic Mottle Virus (CCMV),^68^ and Sindbis Virus,^69^ whose structures have been recently analyzed by Rochal and co-workers^12^ within their proposed scheme. Since these units are asymmetric they appear in multiples of 60 in each of these capsids. Example parameters that support these capsids are given in Table 3.

**Table 3:** Example IH Coefficients *c*_*l*,0_ of the Cost Functions for Single-Shell Capsids in Terms of *N* Single-Protein Subunits

name	*N*	*c*_6,0_	*c*_10,0_	*c*_12,0_	*c*_15,0_
STMV	60	2	0	0	0
L-A	120	10	0	0	0
Dengue	180	0	0	10	10
CCMV	180	2	8	0	0
Sindbis	240	3.5	–5	6	0

**Figure 3:**
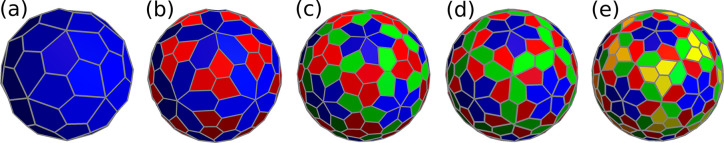
Voronoi representations of global minima from cost functions with the parameters given in Table 3. The structures correspond to the capsids of STMV (a, 60 proteins, ⟨2,1⟩), L-A Virus (b, 120 proteins, ⟨3,1⟩), Dengue Virus (c, 180 proteins, ⟨3,2⟩), CCMV (d, 180 proteins, ⟨4,1⟩), and Sindbis Virus (e, 240 proteins, ⟨4,2⟩). Different orbits of equivalent polygons are shown with different colors.

The capsids are illustrated in Figure 3 using Voronoi tessellation diagrams. The structures in (b) and (c) are anomalous in terms of the CK rules. The others have triangulation numbers *T* = 1 (a), *T* = 3 (d), and *T* = 4 (e). Next, we will show how the introduction of holes can reduce the complexity of the cost function for some of these structures. A hole in our context is a symmetric entity that represents the absence of a real particle. Some capsids may be represented more efficiently in terms of holes and particles than in terms of particles alone, presenting a choice of alternative approaches. Specifically, (1) we could optimize a cost function for the holes and later distribute the particles with a second cost function using electrostatic repulsive Thomson interactions with the holes or (2) define a single cost function for two (three in case e) shells with a common radius, one (two) for the holes and the other one for the particles. In both cases the complexity of the problem is reduced. We discuss the first approach for specificity. Cases (a)–and (c) in Figure 3 are equivalent in terms of holes. Just 12 holes are needed for these capsids, and only the Thomson contribution is required in the cost function to position them correctly. This problem is equivalent to the distribution of the pentamers in a *T* = 1 capsid; thus, the holes appear at the 5-fold sites and their role is therefore the exclusion of the asymmetric protein units from these positions. Cases (d) and (e) require 32 and 42 holes, respectively, in order to exclude proteins from the 5-fold and 3-fold symmetric sites in case (d) and from the 5-fold and 2-fold sites in case (e). The construction followed by Rochal and co-workers to account for all these structures^12^ explicitly excludes proteins from all these sites. The correct arrangement of the 32 holes in case (d) can be obtained with just the Thomson cost function. This problem is equivalent to the location of the 32 capsomers in a *T* = 3 capsid. However, the correct location of 42 holes in case (e) requires a more complex cost function, with either three IH terms in the external potential, or a double shell (with common radius): one with 12 holes and the other one with 30. This problem is equivalent to the distribution of the 42 capsomers in a *T* = 4 capsid, and the same cost function can be used for any of these two problems. Table 4 provides the nonzero example parameters for the two-shell cost function in this case (e), together with the parameters for the trivial cases (a)–(d).

**Table 4:** Example Values for Parameters of One- and Two-Shell Cost Functions Designed to Distribute the Holes Supporting the Structures in Figure 3. *N*_*h*_ Gives the Number of Holes

case	*N*_*s*_	*R*_*s*_	*K*_*s*,*t*_
*N*_*h*_ = 12	12	1	1
*N*_*h*_ = 32	32	1	1
*N*_*h*_ = 42			

As discussed above, the correct distribution of the particles around these fixed holes can now be obtained with a significantly less complex cost function. Table 5 gives the nonvanishing example parameters in the cost function for the capsids in Figure 3. No external potential is required here.

**Table 5:** Parameters for Cost Functions Converting the Holes in Table 4 into Particles, For the Three Cases Given in Figure 3

case	*N*_*s*_	*R*_*s*_	*K*_*s*,*t*_
(c)			
(d)			
(e)			

### Distribution of Combined Subunits in Multiple Shells

**Figure 4:**
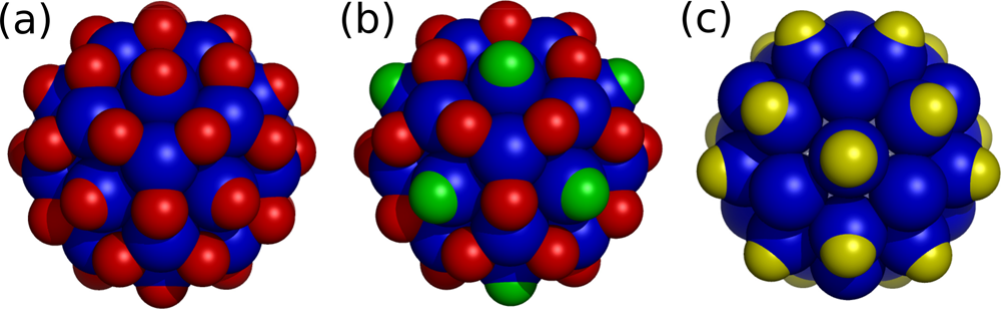
Target structures for the *T*3–*T*4 double capsid. Case (a) corresponds to the cost function with external fields for each shell and the parameters given in Table 6. Case (b) is obtained with the three-shell cost function whose parameters are in Table 7, without external fields. The second coat has two classes of particles (in red and green). Case (c) corresponds to the cost function with 20 holes in the second shell (in yellow).

Two-coat capsids are considered in this subsection, and for capsid building blocks we will employ both capsomers and single proteins. We start by analyzing a two-shell structure that does not yet have a known realization in a natural virus, but this example allows us to summarize the strategies used to find the simplest forms of the cost function defined above using holes or particles of different types. The target structure is a double capsid with an inner *T* = 3 shell containing 32 capsomers, and an outer *T* = 4 coat with 42 capsomers. For this *T*3–*T*4 structure, we start with the most general form of the cost function that includes the two given shells and external potentials for the particles in each one of them. Table 6 provides example parameter values in this case. The global minimum structure supported by the cost function with these parameters is case (a) in Figure 4.

**Table 6:** Example Parameters for a Double-Shell Cost Function with External Fields Supporting the *T*3–*T*4 Target Structure

*N*_*s*_	*R*_*s*_	*c*_*s*,*l*,0_
		

A second choice for the cost function includes three shells, namely, a first inner shell with 32 particles and two common-radius outer shells with 30 and 12 particles, respectively. This model does not require external potentials; example values for the nonvanishing parameters are given in Table 7. Panel (b) in Figure 4 presents the corresponding global minimum obtained with this method and shows the two classes of particles in the outer shell in different colors. As for the first choice, the third approach for this *T*3–*T*4 structure is based on a two-shell cost function with 32 particles in the inner shell and 20 holes in the outer shell interacting attractively with the particles in the inner shell. No external potentials are required in the design of this cost function, and example parameters are given in Table 8. Panel (c) in Figure 4 shows the particle-hole structure obtained with this method. The holes in the outer coat can be converted into particles using a hole-particle cost function with holes fixed at the positions given above and the 32 required particles located in the same shell; holes and real particles interact repulsively. Again, external fields are not required, and example parameters are given in Table 9. The final structure is, of course, the one shown in Figure 4(a).

**Table 7:** Example Parameters for a Three-Shell Cost Function Supporting the *T*3–*T*4 Target Structure

*N*_*s*_	*R*_*s*_	*K*_*s*,*t*_
		

**Table 8:** Example Parameters for a Two-Shell Cost Function with Holes in the Outer Shell Supporting the *T*3–*T*4 Target Structure

*N*_*s*_	*R*_*s*_	*K*_*s*,*t*_
		

**Table 9:** Example Parameters for a Two-Shell Cost Function to Convert the 20 Holes into 42 Particles in the Outer Coat Supporting the *T*3–*T*4 Target Structure

*N*_*s*_	*R*_*s*_	*K*_*s*,*t*_
		

An important feature of the *T*3–*T*4 structure is that the two shells are incommensurate, which probably explains why it is not observed in Nature. Commensurability can be recovered by removing either the red (dimers) or green (pentamers) particles in Figure 4(b). Many *T*3 capsids with protruding symmetric decorations of both types exist.

**Figure 5:**
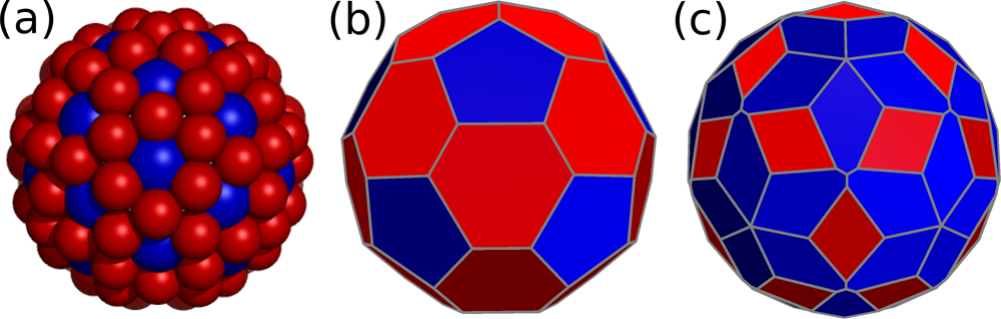
Murine Norovirus illustrations. (a) shows the commensurability of the first and second shell capsomers. (b,c) Voronoi representations of the first and second shells; different colors correspond to polygons in different orbits.

**Figure 6:**
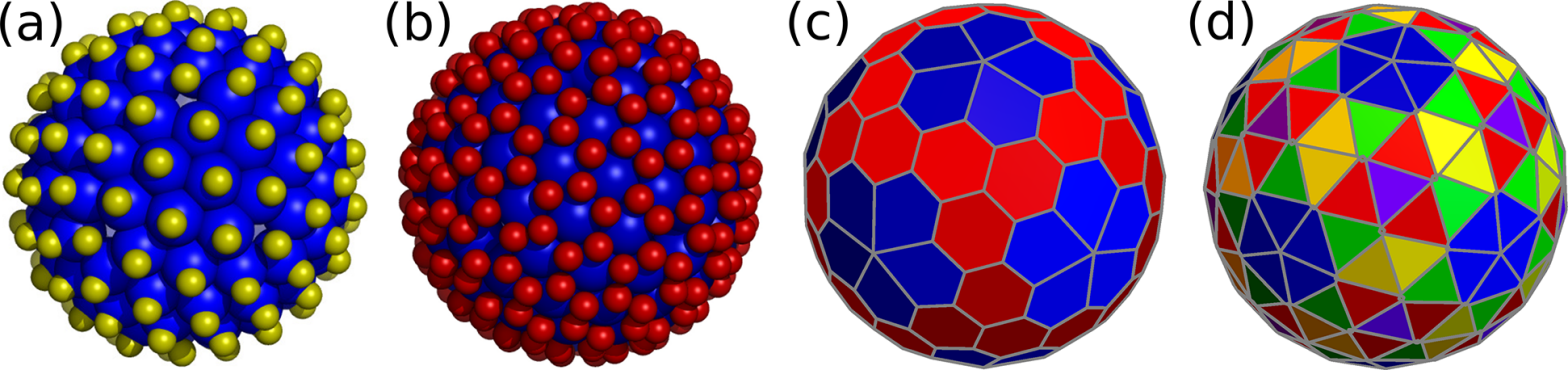
Rice Dwarf Virus illustrations. (a) Commensurability of the first and second shell of proteins (red) and holes (yellow), respectively. (b) Particles conjugate to the holes of the second shell (the trimers). (c,d) Voronoi representations of the first and second shells; different colors correspond to polygons in different orbits.

We now analyze the known double-shell viral capsids of two viruses: the Murine Norovirus (MN)^70^ and the Rice Dwarf Virus (RDV).^71^ These structures have been interpreted by Rochal and co-workers using their construction rules.^7,9−12^ Unlike the previous *T*3–*T*4 capsid, these two capsids feature double-shell commensurability. MN has a *T* = 3 inner shell with 12 pentamers and 20 hexamers and a set of 90 protruding dimers. A simple two-shell cost function can be designed for this virus, with 32 particles in the inner shell and 90 particles in the outer shell, and all the interactions are repulsive. No additional potentials are required. The structure of the double-coat capsid obtained with these cost functions is shown in Figure 5. An even simpler cost function consists of an outer shell with 32 holes interacting attractively with the 32 particles in the inner shell and no external fields. The role of the holes in this case is the exclusion of dimers from the incompatible 5-fold and 3-fold sites (a total of 12 + 20 = 32 sites). On the other hand, the Rice Dwarf Virus capsid has 120 proteins in the inner shell and 260 trimers on the outer shell (Figure 6). A double-shell cost function with these particles requires a large number of terms in the expansions of the external fields, thus becoming quite complex. However, if instead of 260 particles the corresponding 132 holes are used in the second shell, no external fields are required. In this case the hole positions correspond to the capsomer locations of a *T* = 13 CK capsid. These holes can be converted into the 260 particles with another simple two-shell cost function without external fields. Example parameters for these two-shell capsids are provided in Tables 10 and 11.

**Table 10:** Example Cost Function Parameters for MN and RDV Viruses^a^

case	*N*_*s*_	*R*_*s*_	*K*_*s*,*t*_
MN			
RDV			

a MN units are all particles, while RDV units are particles for the inner shell and holes for the outer one.

**Table 11:** Example Parameter Values for the Conversion of Holes into Particles for the Second Shell in RDV

*N*_*s*_	*R*_*s*_	*K*_*s*,*t*_
		

### Polyhedral Designs of Combined Subunits in a Single Shell

In this last subsection, we present cost functions whose targets are capsids that follow the construction scheme proposed by Twarock and Luque.^8^ Our choice includes the smallest capsid derived from the folding of any of the three nontrivial Archimedean hexagonal lattices, namely the trihexagonal, the snub hexagonal, and the rhombitrihexagonal lattice, with triangulation numbers denoted as *T*_*t*_(*h*,*k*), *T*_*s*_(*h*,*k*), and *T*_*r*_(*h*,*k*), respectively. The smallest capsid in each family corresponds to *h* = 1 and *k* = 0. The cost function includes five particles for each pentagonal face and another particle for each one of the triangular (trimers) or rectangular (dimers) faces. Therefore, particles may represent triangular or rectangular units. Holes are also required to reproduce these structures.

**Figure 7:**
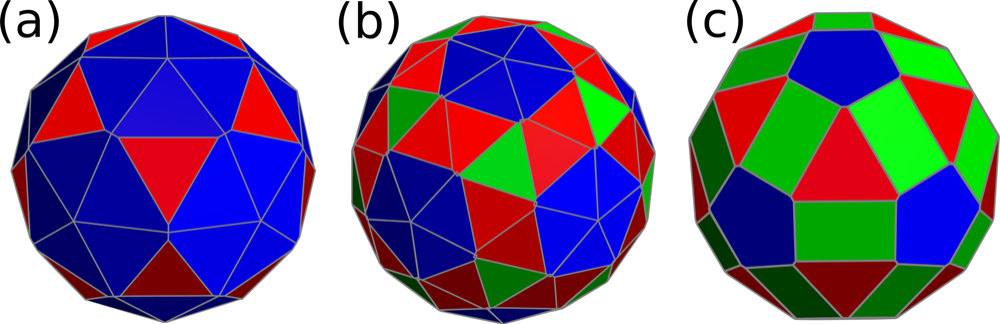
Voronoi representations of the smallest capsids obtained by folding the trihexagonal (a), the snub hexagonal (b), and the rhombitrihexagonal lattice (c); different colors correspond to polygons in different orbits.

We start from the trihexagonal ⟨1,0⟩ capsid with 80 particles and 42 holes. In this case, particles are all triangular units and holes correspond to vertices shared by either five or six triangular faces. We first set up a cost function to fix the 42 holes (30 in 2-fold sites and 12 in 5-fold sites). This problem is equivalent to the location of the capsomers of a *T* = 4 capsid and requires two types of particles (holes in this case) and no external fields. Thus, the cost function is already known (Table 4, *N* = 42). A second cost function with identical repulsive electrostatic interactions among the identical 80 particles and with the fixed holes provides the structure shown in Figure 7a. Its triangular faces split into two orbits.

The snub hexagonal ⟨1,0⟩ capsid requires 72 holes and 140 particles. As in the previous structure, all particles are triangular units and holes correspond to vertices shared by either five or six triangular faces. The hole locations are in one-to-one correspondence with the capsomer positions of a *T* = 7 CK capsid. A simple Thomson cost function distributes these holes correctly. With holes fixed, a second cost function with just one type of particle and equal Thomson repulsion between particles and with the holes gives the target structure shown in Figure 7b. Its triangular faces split into three orbits.

Finally, the rhombitrihexagonal ⟨1,0⟩ capsid would require 72 holes and 110 particles. At this resolution, the appropriate cost function is relatively complex and must separate holes and particles in different sets and include external fields. Instead, we can increase the coarse-graining by joining the five particles in each pentagonal face into a pentameric building block to produce a simpler cost function. This function must either use an external field with the contribution of the first three IH, or separate capsomers into three shells (same radius): 12 pentamers, 20 trimers and 30 dimers. For the latter choice only Thomson interactions are required, with example parameters given in Table 12. The corresponding target structure is given in Figure 7c. Each capsomer type defines an orbit.

**Table 12:** Example Parameters for the Three-Shell Cost Function Designed for the Rhombitrihexagonal Capsid Shown in Figure 7c

*N*_*s*_	*R*_*s*_	*K*_*s*,*t*_
		

Possibly due to their complexity, structures (b) and (c) in Figure 7 have not been observed in Nature.

## Conclusions

The main objective of this work was the derivation of minimal complexity design functions whose global minima are the observed structures of icosahedral viral capsids to uncover the simplest conditions on the interparticle interactions that support these structures. It is obvious that the information content and complexity of these functions are directly related to the number of particles in the target structure and the number of required parameters in the cost function. Is there a more precise way to define those properties? The answer is formally yes: we just have to identify the number of structurally different global minima *N*_g_ in the parameter space of the given cost function for fixed particle numbers. This space includes the other parameters in  that have not been scaled to one or fixed to zero. We identify each one of the distinct global minima of  with a possible target state of the cost function. If we assign equal weights to all of them then the information content measure of the cost function is *S*_cf_ ∼ log(*N*_g_). This measure corresponds to the maximum Shannon entropy for a system with *N*_g_ states,^72^ which in this case is known to be directly related to the Kolmogorov complexity.^73^ Two examples serve to illustrate the use of this complexity measure: the *T* = 3 and *T* = 4 capsids at the capsomer scale. The *T* = 3 capsid is the target structure of a Thomson cost function with 32 particles, and no adjustable parameters, so there is a single global minimum (*N*_g_ = 1, *S*_cf_ = 0). In contrast, the *T* = 4 cost function requires two sets of particles (12 pentamers [p] and 30 hexamers [h]) with two adjustable interaction parameters *K*_h,h_ and *K*_p,h_ (*K*_p,p_ = 1 after scaling). In this two-dimensional parameter space one can identify at least two different structures: if *K*_h,h_ = *K*_p,h_ = *K*_p,p_ = 1, one recovers the Thomson problem for 42 particles where the global minimum is not icosahedral but has point group *D*_5*h*_.^55,56^ However, if *K*_h,h_ ≃ *K*_p,h_ ≃ 0.1 the global minimum corresponds to the *T* = 4 structure. There are other possible global minima, and therefore *N*_g_ > 2. We conclude that the complexity *S*_cf_ for *T* = 4 is higher than for *T* = 3. *N*_g_ depends on both the particle and parameter numbers and it would require a systematic search of parameter space to identify all the possible global minima. When the number of parameters increases the complexity cannot fall. We also expect the complexity to rise with the number of particles, except perhaps at magic number sizes, when a particularly advantageous packing exists, which may extend over a relatively wide range of parameter space.

Another relevant issue concerns the complexity of the target structures themselves. Consider, for instance, an asymmetric distribution of 12 identical particles on the sphere. It is clear that one has to give the location of at least 11 of them (with respect to the other one) to provide the structure. However, if the 12 particles are at the vertices of an inscribed icosahedron then the position of just one particle and its equivalence with the other ones by symmetry is the only required information. From the Kolmogorov perspective the latter geometry is simpler. A precise measure of this complexity *S*_ts_ may be formulated using the concept of orbits and the permutation group. For the asymmetric structure the number of orbits is equal to the number of particles; all particles are inequivalent and all permutations correspond to different structures. The number of inequivalent permutations is therefore 12! and the corresponding maximum entropy of this geometry *S*_ts_ ∼ log(12!) (*S*_ts_ ∼ log(*N*!), in general). In the case of the icosahedral geometry, the 12 equivalent particles define an orbit; all permutations correspond to the same structure and *S*_ts_ ∼ log(1) = 0 in this case if the particles are indistinguishable. This idea is straightforwardly generalized to more than one orbit, when only permutations between inequivalent particles in different orbits are identified as different structures. From this viewpoint the larger the number of orbits and particles in a given structure, the more complex it is.

The structural properties of icosahedral virus capsids compatible with a simple description are their symmetry and the uniform distribution of the constituent building blocks, which follow the pattern of optimal packing. Exceptions to this optimal packing arise when intrinsically different subunits exist or subunit symmetry constraints prevent particles from being located at certain positions. For example, proteins, as asymmetric building blocks, cannot be placed on a symmetry element, such as a rotation axis. In our design function this prohibition can be accomplished by the use of external fields or, in a simpler way, by employing holes. Furthermore, holes may be present in real capsids, where they may be associated with a biological function. In this case the construction of a simple cost function will probably require consideration of holes as possible building blocks.

In the cost function design, optimal packing has been incorporated through the Thomson model. Symmetry constraints have been included, with external fields written as reduced expansions in the basis set of icosahedral harmonics. The simplest cost functions were obtained by associating particles in disjoint inequivalent sets with distinct interactions, or by introducing interacting holes corresponding to the absence of particles. We have obtained relatively simple cost functions for a wide variety of single-shell and double-shell icosahedral capsids, suggesting that this approach is an effective design tool.

The two complexity measures introduced in this work allow a classification of icosahedral capsids in terms of their complexity. In agreement with previous analyses,^74^ complexity increases with the number of orbits in the final structure, but additional factors that affect complexity are revealed in the design. We have given some illustrations of the connections between these complexities and considerations such as natural abundance and knowledge of the essential components of the interparticle potential required to support these target structures as ground states. Let us recall, for instance, the comparative analysis of the complexities of *T* = 3 and *T* = 4 capsids at the capsomer scale. Both structures have the same number of orbits (just 2) and therefore similar structural complexity *S*_ts_. However, while the *T* = 3 capsid has the simplest Thomson form for the cost function, the *T* = 4 example requires either an external field or two kinds of particle, which implies a significant increase in the complexity measure *S*_cf_. This observation is consistent with the difficulty found in previous coarse-grained interaction models to reproduce the *T* = 4 capsid,^16,31,47^ and the relative numbers of *T* = 3 and *T* = 4 known in Nature. Similar increased cost function complexity is generally required for all the ⟨*h*,0⟩ structures.

The most complex capsids targeted in this work are the polyhedral designs proposed by Twarock and Luque^8^ derived from the snub hexagonal and the rhombitrihexagonal lattices. No naturally occurring capsids are known to follow these constructions, and thus, the increased complexity does not seem to provide any fitness improvement or evolutionary advantage. Another example of these connections has been provided, at the capsomer scale, for *T* = 7 capsids, which appear as the global minimum of the simplest Thomson cost function. This cost function and the particular location of the capsomers in this capsid suggest that they could be built from just one type of capsomer with 5-fold symmetry (pentamers). The papillomavirus capsid^60^ is a realization of this construction.

## Methods

**Figure 8:**
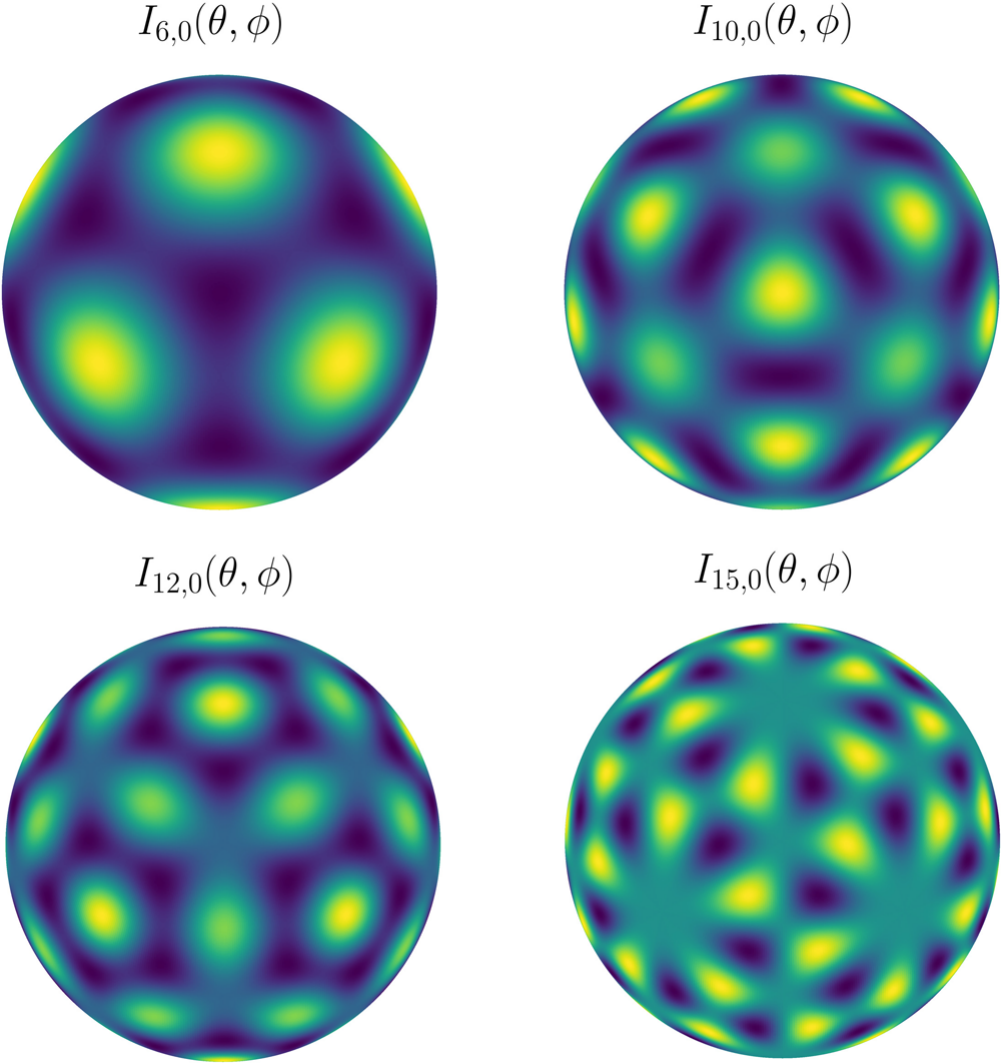
Density plots of the first four nontrivial IH.

The external fields in the cost functions are expanded in a basis set of icosahedral harmonics *I*_*l*,*n*_(θ,ϕ). These are linear combinations of spherical harmonics of the same order *l*, which transform as the totally symmetric irreducible representation in icosahedral symmetry. Values for *l* are restricted to a sparse subset of the positive integers and *n* takes integer values from 0 to a maximum *n*_*l*_ – 1, with *n*_*l*_ = 1 up to *l* = 30. Properties and explicit forms of these *I*_*l*,*n*_(θ,ϕ)are given by Zheng and Doerschuck.^57^ We have used these forms in our scheme. Figure 8 illustrates the first four nontrivial IH, which are the most useful for our applications. With just the first three IH one can favor or disfavor selectively the presence of particles at the positions of each type of rotation symmetry axis (2-fold, 3-fold, or 5-fold). *I*_15,0_ is the first odd IH and can be used to break inversion symmetry.

The global minimum of  in the landscape of particle configurations was found using basin-hopping global optimization.^58,59^ Here, we employed four different runs with between 100 and 1000 basin-hopping steps. We observed that the choice of the basin-hopping temperature parameter was not crucial, and values spanning an order of magnitude worked almost equally well. No competing structures were observed, indicating that the parameter space for these cost functions leads to strongly funneled landscapes.^75−78^
